# Production of blueberry wine and volatile characterization of young and bottle‐aging beverages

**DOI:** 10.1002/fsn3.895

**Published:** 2019-01-28

**Authors:** Ana Mendes‐Ferreira, Eduardo Coelho, Catarina Barbosa, José M. Oliveira, Arlete Mendes‐Faia

**Affiliations:** ^1^ Department of Biology and Environment WM&B – Wine Microbiology & Biotechnology Lab University of Trás‐os‐Montes and Alto Douro Vila Real Portugal; ^2^ BioISI—Biosystems & Integrative Sciences Institute Campo Grande Portugal; ^3^ CEB–Centre of Biological Engineering University of Minho Braga Portugal

**Keywords:** alcoholic fermentation, blueberry wine, bottle‐aging, GC‐FID, GC‐MS, volatile compounds

## Abstract

The aim of this study was the production of blueberry wine and the characterization of the volatile compounds of fermented and aging in bottle products. Multivariate data analysis indicated similarity of volatile compounds released when fermentations were conducted at laboratory‐scale and midscale, with the exception of one replicate creating a distinctive group characterized by low concentrations of acetaldehyde, methanol, 1‐hexanol, and ethyl hexanoate, and the production of polyalcohols such as 2,3‐butanediols. This experiment was the only one where no adjustments of *YAN* were performed. Some of the major volatile compounds (acetaldehyde, ethyl acetate, 2‐methyl‐1‐butanol, 3‐methyl‐1‐butanol, and 2‐phenylethanol) were found above their perception thresholds. Esters and terpenic compounds were the groups of volatiles expressed the most in blueberry wines, followed by volatile fatty acids, alcohols, and norisoprenoids (3‐hydroxy‐7,8‐dihydro‐β‐ionone, 3‐oxo‐α‐ionol, and 3‐hydroxy‐7,8‐dihydro‐β‐ionol). The wines that experienced bottle‐aging are characterized by high concentrations of ethyl esters, diethyl succinate, ethyl lactate, and diethyl malonate. The results contribute for deeper knowledge of the technological procedure, analytical characteristics, and volatile compounds of blueberry wines, reinforcing the interest in this beverage and opening perspectives for further studies on the production of new blueberry‐based products with differential characteristics that value its nutraceutical and functional properties.

## 1 INTRODUCTION

The production and consumption of blueberries (*Vaccinium corymbosum* L.) has remarkably increased worldwide in recent years, mainly driven by their reported nutraceutical functions, human health benefits due to their noticeable antioxidant capacity (Prior et al., 1998) (for anti‐aging and degenerative disease prevention), high contents of vitamins and minerals, and low calorie content (Giongo et al., 2011).

Blueberry crop production in Portugal started in the 1990s in Sever do Vouga, where its cultivation quickly turned into an added‐value product for the family economy. In 2007, approximately 95 % of the production was exported and placed on the European market, earlier than that of other competitors (Serrado, Pereira, Freitas, Martins, & Dias, 2008). The blueberry market continued to grow, and in 2015, the export value increased to almost one million euros, with the expectation of this amount doubling by the end of 2017. Blueberries are mainly commercialized as fresh fruits, but with the expansion of this highly perishable fruit, farmers have tried to find other strategies to preserve it. In addition to the freezing of blueberries, the transformation of blueberries into juices, jellies, and powders is still an option that guarantees the availability of this fruit in the market, keeping the essential bioactive compounds so desired by consumers (review in Michalska & Łysiak, 2015). In fact, comparative studies demonstrate that the antioxidant activity of anthocyanin extracts from blueberries is not significantly altered in fresh, dried, or frozen blueberries (Lohachoompol, Srzednicki, & Craske, 2004). The high antioxidant activity of these fruits has been correlated with health‐promoting benefits, conferring the title of functional food to blueberries (Huang, Zhang, Liu, & Li, 2012). These properties mainly result from the high content of phenolic compounds, including anthocyanins, chlorogenic acids, flavonols, and procyanidins (Kim, Kim, Kim, & Park, 2013). The major flavonoids in berries and red grapes are anthocyanins and flavonols, mainly present in glycosylated forms (Cho, Howard, Prior, & Clark, 2004). The ranges of total anthocyanin and total flavonol contents (expressed in mg/kg) varied in blueberries from 1435.2 to 8227.3 and from 172.5 to 327.5, respectively, and in red grapes from 380.9 to 7904.7 and from 21.0 to 322.2 (Cho et al., 2004). Similarly, in red grapes, anthocyanins are mainly present in the skins (Riihinen, Jaakola, Ka¨renlampi, & Hohtola, 2008), while tannins and phenolic acids predominate in seeds (Santos et al., 2016). In addition to phenolic compounds, ripe blueberries also contain sugars, but their average content is half of that found in grapes, that is, usually less than 100 g/kg of fresh fruit (Kalt & McDonald, 1996), with glucose and fructose being the greatest percentage of total soluble carbohydrates (Skrovankova, Sumczynski, Mlcek, Jurikova, & Sochor, 2015) without sucrose (Kalt & McDonald, 1996). In a completely different location, sugar content (°*Brix*) ranged from 8.3 to 14.30 in six different cultivars (Kim et al., 2013), indicating that the amount of sugars is dependent on the cultivar, the degree of ripeness, and location. Thus, total sugar content can be a major criterion for ripeness and consequently for selecting the optimal harvesting point. The organic acids reported in blueberries are citric, malic, succinic, and quinic acids, and the two studied species are distinguishable based on their acid profiles (Ehlenfeldt, Meredith, & Ballington, 1994). Several amino acids other than proline have also been found (Zhang et al., 2014). At normal maturity, an average blueberry fruit consists of approximately 84% water, 0.4% to 0.5% fat, 1.5% to 3.5% dietary fiber, 9.7% carbohydrate, 3.5% cellulose (Keservani, Sharma, & Kesharwani, 2016), 0.7% soluble pectin, several minerals (Zhang et al., 2014), and 0.6% to 0.7% of protein (Michalska & Łysiak, 2015), as mass per mass of fresh fruit. The vitamins in blueberry are vitamin A, niacin, vitamin C (Michalska & Łysiak, 2015), and folic acid (Skrovankova et al., 2015); ascorbic acid shows significant variability between cultivars and species, ranging from 13 to 164 g/kg, with an average of 125 g/kg (Prior et al., 1998), which corresponds to 1/3 of its daily recommended intake (Keservani et al., 2016). In fact, blueberry is considered one of the greatest sources of antioxidants among all fruits and vegetables (Prior et al., 1998). Continuous expansion of blueberry production incentivizes farmers to search for new technological options for added‐value products. Thus, the main goal of this study was to produce an alcoholic beverage from blueberry fruit following the traditional technology used to produce wine and to characterize its volatile composition using gas chromatography‐mass spectrometry (GC‐MS) for the determination of minor compounds and gas chromatography‐flame ionization detection (GC‐FID) for major compounds. Few studies have investigated the production of blueberry wines and the volatile composition of this fruit wine remains poorly understood, although aroma is one of the most important qualities for wine products. We anticipate that this study of blueberry wine processing and the evaluation of the aroma compounds of this beverage can contribute to the additional use of this fruit for the production of “fruit wines” or other derivatives.

## 2 MATERIALS AND METHODS

### 2.1 Strain and inoculum preparation

*Saccharomyces cerevisiae* Lalvin QA23 (Lallemand, Montreal, Canada) was used in this study as an active wine dry yeast. Starter cultures were prepared by rehydration of the active dry yeasts according to the manufacturer's instructions to obtain cell populations (as colony‐forming units—*CFU*) of 10^8^ ml^−1^. This preculture was used to inoculate blueberry juice with an initial cellular concentration of 10^6^ ml^−1^.

### 2.2 Preparation of blueberry juice for fermentation

Blueberry wine was produced from *Vaccinium corymbosum* “Duke,” acquired from Cuvelo, Vila Pouca de Aguiar, in the northeastern region of Portugal, from a local farmer who produces approximately 7 t of fruit and exports 60 % of it as fresh berries. In this farm, blueberry fruits are grown in accordance with the principles of organic farming. After harvest, the fruits were kept frozen until the beginning of the experiments. Thus, immediately before the trials, the fruit was thawed, selected, and manually pressed for must preparation. Sulfur dioxide, in a 6% solution, was added up to a concentration of 50 mg/L to prevent unwanted microbial growth. Then, the juice was analyzed to determine the following parameters: º*Brix*, pH, titratable acidity (*TA*), and yeast assimilable nitrogen (*YAN*). Based on the results, adjustments of sugar, *TA,* and nitrogen content were performed to optimize yeast performance during alcoholic fermentation. *TA*, as tartaric acid, was adjusted to 3.5 g/L with L‐(+)‐tartaric acid (Panreac, Spain). The nitrogen content, as *YAN*, was adjusted to 120 to 140 mg/L with diammonium hydrogen phosphate (DAP—Merck, Germany). To obtain an alcoholic beverage with an alcoholic strength (by volume) of approximately 11 %, blueberry–juice º*Brix* was corrected to 20. The parameters º*Brix*, pH, *TA,* and *YAN* concentration were determined after the adjustments.

**Figure 1:**
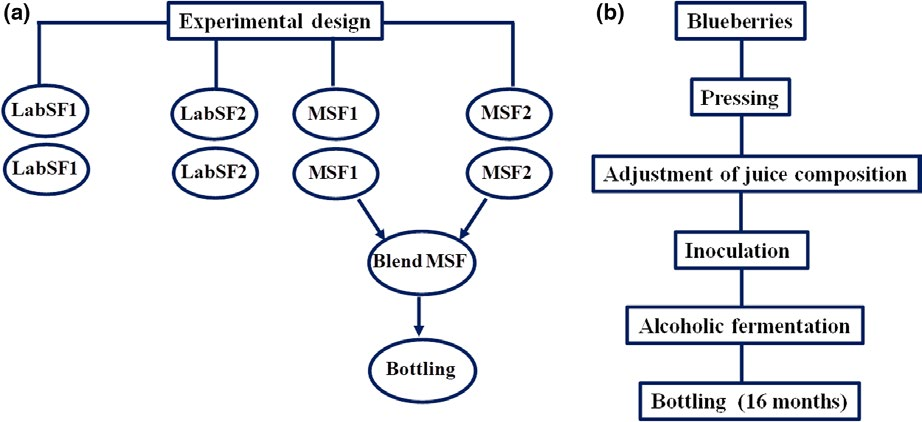
Flowchart showing the experimental design (a) and the major steps of blueberry wine production (b)

Insoluble solids maintained contact with the fermented juice until 20 to 30 g (1.020 of specific gravity) of sugar remained. At the end of alcoholic fermentation, unclarified juice was centrifuged at 10,000 *g* for 10 min in a Sorvall centrifuge and kept in a refrigerator until posterior analysis. All assays were conducted in duplicate and repeated two more times as biological replicates. The experimental design and the flowchart of blueberry wine production are summarized in Figure 1.

### 2.3 Fermentation assays

#### 2.3.1 Laboratory‐scale fermentations (LabSFs)

The mixture was subdivided into 1‐L flasks filled to 2/3 of their volume and inoculated with the selected wine yeast strain. Two fermentations were conducted at approximately 22 to 25°C without shaking in the presence of 2/3 of the skins (LabSF 1 and LabSF 2). Fermentations were monitored daily by weight loss as an estimate of CO_2_ production.

#### 2.3.2 Midscale fermentations (MSFs)

Another two fermentations were performed later using the same procedure in 50‐L fermenters of stainless steel (MSF 1 and MSF 2). In sum, fruit berries were pressed in a manual vertical press, and after the addition of SO_2_ (50 mg/L), juice composition was adjusted based on the results previously obtained under laboratory conditions. Alcoholic fermentation was conducted in contact with all the skins. Fermentation was monitored daily by determination of temperature and specific gravity; when the specific gravity reached 1.020, the fermented juice was removed from the skins, and the fermented product was kept separately in full 20‐L glass bottles until the end of total sugar breakdown.

At the end of alcoholic fermentation, samples were taken from all fermented media to determine the production of minor volatile compounds by GC‐MS and major compounds by GC‐FID.

#### 2.3.3 Post‐fermentation wine handling, bottling, and aging

At the end of alcoholic fermentation, the temperature of the wines MSF 1 and MSF 2 was decreased to 4°C, and the yeast was allowed to settle for 10–15 days. Sulfur dioxide was added to achieve a concentration of 15 to 20 mg/L of free sulfur dioxide. To investigate the effects of aging on volatile composition, a blend of MSF 1 and MSF 2 wines was made. Blended wines corresponded to the two replicates of MSF 1 and the two replicates of MSF 2. These wines consisted of equal proportions of MSF 1 and MSF 2 beverages. The blended wine was bottled in 100 small bottles of 350 ml volumes and kept at approximately 18°C in a cellar. After 16 months, two bottles were randomized and opened for chromatographic analysis.

### 2.4 Analytical determinations

Total and free SO_2_, pH, titratable acidity (*TA*), volatile acidity (*VA*), and alcoholic strength by volume (*AS*_v_) were determined according to validated standard methods (OIV, 2015). *YAN* was determined by the formaldehyde method as previously described (Aerny, 1996). All physicochemical analyses were performed prior to and at the end of alcoholic fermentations.

### 2.5 Organic acids and glycerol

Organic acids and glycerol were quantified by high‐performance liquid chromatography (HPLC Flexar, PerkinElmer, Shelton, Connecticut, USA) using a system equipped with an ion exchange column (Aminex HPX‐87H, Bio‐Rad Laboratories, Hercules, CA, USA) and refractive index and UV/Vis detectors. The eluent was sulfuric acid (5 mmol/L) at a flow rate of 0.6 ml/min. The column was maintained at 60°C, and a sample volume of 6 μl was injected. The components were identified, using the PerkinElmer Chromera Software, through their retention times relative to respective standards. Citric acid was spectrophotometrically quantified using an enzymatic assay procedure (Y15, © Biosystems S.A., Barcelona Spain).

### 2.6 Analysis of volatile compounds

#### 2.6.1 Major compounds

Major volatiles were analyzed using a CP‐9000 chromatograph (Chrompack) equipped with a Meta‐WAX capillary column (30 m × 0.25 mm; 0.20 μm film thickness) and a flame ionization detector (FID). Helium was used as the carrier gas with an initial flow rate of 1 ml/min. Samples were mixed with 4‐nonanol as an internal standard at a final concentration of 60 mg/L, and 1 μl of mixture was injected. The temperatures of the injector and detector were both maintained at 250°C. The column was initially at 50°C, then heated to 177.5°C at a rate of 5°C/min and finally heated to 230°C at 10°C/min, which was held for 15 min. Quantification was performed using Star Chromatography Workstation version 6.41 (Varian) software with response factors and retention times determined with pure standards.

#### 2.6.2 Minor compounds

Minor volatiles were analyzed by GC‐MS after extraction of 8 ml samples with 400 μl of dichloromethane, using 4‐nonanol as an internal standard (final concentration of 300 μg/L). A Varian 3800 gas chromatograph equipped with a 1079 injector and a Varian Saturn 2000 ion‐trap mass spectrometer were used. Each 1 μl injection was made in splitless mode (30 s) in a Sapiens‐Wax MS column (30 m × 0.15 mm; 0.15 μm film thickness, Teknokroma). The carrier gas was helium 49 (Praxair) at a constant flow rate of 1.3 ml/min. The detector was set to electron impact mode with an ionization energy of 70 eV, a mass acquisition range (*m*/*z*) from 35 to 260, and a 610‐ms acquisition interval. The oven temperature was initially set to 60°C for 2 min, then raised from 60°C to 234°C at a rate of 3°C/min and from 234°C to 260°C at 5°C/min and finally maintained at 260°C for 10 min. The injector temperature was 250°C with 30 ml/min split flow. Compounds were identified using MS Workstation version 6.9 (Varian) software by comparing mass spectra and retention indices with those of pure standards. Minor volatile compounds were quantified as 4‐nonanol equivalents.

### 2.7 Statistical analysis

The data are presented as the mean values with their standard deviation. One‐way analysis of variance (ANOVA) and comparison of treatment means (LSD, *p* < 0.05) of each volatile compound were performed using Statistica 6.1 software (Statsoft, Tulsa, OK, USA). The Tukey HSD (honest significant difference) test was used for paired comparisons. Differences between the volatile compound profiles of wines were explored using principal component analysis (PCA) performed by JMP 7.0 software (SAS Inc., 2007).

## 3 RESULTS AND DISCUSSION

### 3.1 General physicochemical characterization of blueberry juice and wine

Freshly squeezed blueberry juice of *Vaccinium corymbosum* “Duke” had a relatively high pH and consisted of sugars, which were characterized by a °*Brix* of approximately 13, and a limited concentration of assimilable nitrogen, as displayed in Table 1. The levels of sugars (°*Brix*) are in accordance with the higher values reported for other cultivars in Korea, which ranged from 8.3 to 14.30 (Kim et al., 2013), but are relatively higher than those reported in California, where the mean values ranged from 77.2 to 103.5 g/kg in ripe or overripe blueberries (Kalt & McDonald, 1996), indicating the dependence of this parameter on the cultivar, degree of ripeness, and location. *TA* values obtained herein (1.5–4.0 g/L) are in agreement with results previously reported for lowbush blueberry cultivars (Kalt & McDonald, 1996). In this study, *YAN* ranged from 52.5 to 126 mg/L (Table 1), concentrations similar to those reported in grape berries (from 50 to 450 mg/L). A mean value of 140 mg/L is the value given as sufficient for complete fermentation of reasonably ripened grapes (Agenbach, 1977). One strategy used by technologists for nitrogen‐limited juice is the addition of nitrogen supplements such as inorganic forms like diammonium phosphate prior to grape‐juice fermentation (Mendes‐Ferreira, Mendes‐Faia, & Leão, 2004). Accordingly, to obtain an alcoholic fermentation with an alcoholic strength, by volume, of approximately 11 %, several adjustments were made prior to fermentation: Sucrose was added to attain a ^°^ *Brix* of 20, and assimilable nitrogen, as *YAN*, was adjusted to 140 mg/L with diammonium phosphate in all experiments, with the exception of one set of fermentations that already had an initial concentration of 126 mg/L (LabSF 1). The results of chemical analyses of the juices before and after adjustments of juice composition, as well as of final wines, are presented in Table 1.

**Table 1:** Physicochemical characteristics of blueberry juices and respective wines after alcoholic fermentation by the yeast strain *Saccharomyces cerevisiae* QA23 in laboratory‐scale fermentations (LabSFs) and midscale fermentations (MSFs) or after 16 months in bottle

Juices	Before corrections				After corrections			
	LabSF 1	LabSF 2	MSF 1	MSF 2	LabSF 1	LabSF 2	MSF 1	MSF 2
°*Brix*	13.8	14.2	13.4	10.8	17.4	18.2	18.0	17.8
pH	4.3	4.05	3.892	3.218	3.23	3.3	3.3	3.218
*TA*/(g/L)	1.69	1.5	1.5375	4.05	3.08	3.196	3.1375	4.05
*YAN*/(mg/L)	126	52.5	52.5	66.5	126	152.5	152.5	166.5

Wines	LabSF 1	LabSF 2	MSF 1	MSF 2	Aged in bottle			
pH	3.29 ± 0.05	3.23 ± 0.03	3.22 ± 0.13	3.18 ± 0.20	3.11 ± 0.12			
*YAN*/(mg/L)	0.00 ± 0.00	1.75 ± 3.50	0.00 ± 0.00	13.75 ± 4.60	8.75 ± 2.47			
*AS*_*v*_/%	11.60 ± 0.42	11.02 ± 0.18	11.40 ± 0.00	10.48 ± 0.39	11.20 ± 0.85			
*TA*/(g/L)	5.34 ± 0.73	5.22 ± 0.60	5.58 ± 1.08	6.38 ± 0.75	5.68 ± 0.18			
*VA*/(g/L)	0.48 ± 0.04	0.31 ± 0.07	0.35 ± 0.02	0.26 ± 0.06	0.23 ± 0.02			
*C*_SO2.total_/(mg/L)		51.84 ± 6.34	69.16 ± 0.06	38.50 ± 3.54	60.18 ± 15.39			
*C*_SO2.free_/(mg/L)	* *	1.60 ± 0.00	1.60 ± 0.00	0.80 ± 1.13	4.64 ± 4.30			

*Note*

C_SO2_: Mass concentration of SO_2_; *AS*_v_: Alcoholic strength, by volume; *TA*: Titratable acidity, defined as the sum of the titratable acids at pH 7 by addition of a titrated alkaline solution, expressed as tartaric acid; *VA*: Volatile acidity, expressed as acetic acid; *YAN*: Yeast assimilable nitrogen—is the sum of total amino acids minus proline, expressed as N.

Accordingly, the alcoholic strength by volume ranged from 10.2 % to 11.9 %, slightly different than expected (11.7 %), suggesting that substances other than sugars interfered with the measurement of °*Brix* by refractometry in this type of matrix. Only traces of assimilable nitrogen were found in the final wines, indicating that all the *YAN* was necessary for the strain *S. cerevisiae* Lalvin QA23 to complete sugar breakdown. The pH values of the final fruit wines varied from 3.00 to 3.26, with values very similar to those of the corrected juice. In contrast, *TA* varied between 5.1 and 6.9 g/L, approximately the double of the total acidity of the corrected juices (3.0 to 4.0 g/L) prior to alcoholic fermentation. This increase in acidity can be attributed to the production of succinic acid by yeasts during alcoholic fermentation (Table 2). Other acids determined by HPLC, such as citric and malic acids, were found in this case as only traces rising from fruit berries. Volatile acidity is an enological parameter that refers to the steam‐distillable acids present in wine, primarily acetic acid (approximately 90 % of the volatile acids) but also lactic, formic, butyric, and propionic acids (Boulton, Singleton, Bisson, & Kunkee, 1996; Swiegers, Bartowsky, Henschke, & Pretorius, 2005) in the free state and as salts (OIV – The International Organisation of Vine and Wine, 2015). The average level of acetic acid produced by *Saccharomyces* ranges from 100 to 200 mg/L and is influenced by yeast strain, fermentation temperature, and juice composition (Boulton et al., 1996; Ribéreau‐Gayon, Dubourdieu, Donèche, & Lonvaud, 2006). In blueberry wines, *VA* ranged from 0.22 to 0.51 g/L, in LabSF and MSF, respectively (Table 1). The high value of *VA* cannot be attributed to the development of spoilage bacteria because we did not find lactic acid in the wines and the amount of acetic was similar in all experiments (Table 2). The use of large fermenters probably altered the yeast growth profile and fermentation conditions, which therefore restrained the production of acetic acid (Ugliano & Henschke, 2009). Despite this slight variation, the values are in accordance with those found and reported in wines or in other fermented fruit beverages. Total SO_2_ values in wines were similar to those found in the corrected juices (50 mg/L). The concentration of free sulfur dioxide was practically zero, which is typical considering that SO_2_ can be combined with compounds derived from fruit (sugar, anthocyanin pigments) or with other compounds formed during fermentation by yeast such as acetaldehyde, pyruvate, and keto‐glutarate or other keto‐acids (Boulton et al., 1996). The concentration of glycerol produced by yeast was independent of the fermentation conditions.

**Table 2:** Concentration of organic acids (*C*) in blueberry wines after alcoholic fermentation by the yeast strain *Saccharomyces cerevisiae* QA23 in laboratory‐scale fermentations (LabSFs) and midscale fermentations (MSFs) or after 16 months in bottle

Treatment	Citric acid *C*/(g/L)	Tartaric acid *C*/(g/L)	Malic acid *C*/(g/L)	Succinic acid *C*/(g/L)	Lactic acid *C*/(g/L)	Acetic acid *C*/(g/L)	Glycerol *C*/(g/L)
LabSF	1.26 ± 0.15	3.68 ± 0.36	0.11 ± 0.16	2.80 ± 1.66	nd	0.18 ± 0.00	5.19 ± 1.23
MSF	2.26 ± 0.15	5.86 ± 3.67	0.16 ± 0.08	4.80 ± 0.04	0.05 ± 0.07	0.22 ± 0.07	5.16 ± 1.28
Aged in bottle	1.77 ± 0.024	3.53 ± 0.27	nd	2.34 ± 0.05	nd	0.24 ± 0.10	5.03 ± 0.24

*Notes*

The results are shown as the mean values with their standard deviation.

nd: not detected.

### 3.2 Volatile aroma compounds in blueberry wines

#### 3.2.1 Major volatiles

A mixture of major volatile compounds was identified in blueberry “wines,” and these compounds included acetaldehyde, ethyl acetate, methanol, 1‐propanol, 2‐methyl‐1‐propanol, 2‐methyl‐1‐butanol, 3‐methyl‐1‐butanol, 2,3‐butanediol, and 2‐phenylethanol, as displayed in Table 3. These compounds are derived mainly from yeast metabolism and participate in the aromatic quality of wine (Ribéreau‐Gayon et al., 2006). Some major volatiles were found above the described perception threshold, namely acetaldehyde, ethyl acetate, 2‐methyl‐1‐butanol, 3‐methyl‐1‐butanol, and 2‐phenylethanol. Acetaldehyde in blueberry wines surpassed the concentrations typically observed in wine (10–75 mg/L) (Bartowsky & Pretorius, 2009). Acetaldehyde constitutes approximately 90 % of the total aldehyde content in wine; it can be formed by yeasts and acetic acid bacteria or as a result of the auto‐oxidation of ethanol and oxidation of phenolic compounds (Liu & Pilone, 2000). This carbonyl compound also contributes to the persistence of the color in red wine (Liu & Pilone, 2000) and, when present in excess, imparts an undesirable green, grassy, apple‐like aroma (Osborne, Mira de Orduña, Pilone, & Liu, 2000; Swiegers et al., 2005). Acetaldehyde was the major volatile, and its concentration decreased the most during aging in bottles. In wine, the decrease in acetaldehyde concentration after alcoholic fermentation can be associated with the occurrence of malolactic fermentation (Osborne et al., 2000), condensation with wine phenolic compounds and reduction to form acetoin and 2,3‐butanediol (Zea, Serratosa, Mérida, & Moyano, 2015). Higher alcohols are produced by yeasts from branched‐chain amino acids via the Ehrlich pathway or from catabolic sugar metabolism. In this study, the alcohols, particularly 3‐methyl‐1‐butanol, are quantitatively the largest group of volatile compounds, and 3‐methyl‐1‐butanol was detected in all blueberry wines at similar concentrations, ranging from 180.26 to 222.95 mg/L, a desirable level for giving complexity to wine (Swiegers et al., 2005; Ugliano & Henschke, 2009).

**Table 3:** Concentration of major volatile compounds (*C*) detected and quantified by GC‐FID in blueberry wines after alcoholic fermentation by the yeast strain *Saccharomyces cerevisiae* QA23 in laboratory‐scale fermentations (LabSFs) and midscale fermentations (MSFs) or after 16 months in bottle

Compounds *C*/(mg/L)	LabSF 1	LabSF 2	MSF 1	MSF 2	Aged in bottle
Acetaldehyde	101.72 ± 1.72	169.21 ± 33.55	225.29 ± 1.05	159.57 ± 54.57	112.42 ± 4.11
Ethyl acetate	42.13 ± 0.01	39.62 ± 2.99	53.75 ± 3.34	48.24 ± 10.10	33.41 ± 2.28
Methanol	174.89 ± 12.74	275.56 ± 10.92	281.72 ± 28.34	367.33 ± 75.14	284.04 ± 69.28
1‐Propanol	15.19 ± 0.66	21.10 ± 0.65	21.84 ± 2.09	30.49 ± 3.35	27.48 ± 0.91
2‐Methyl‐1‐propanol	78.88 ± 2.64	26.51 ± 0.73	45.62 ± 4.79	72.99 ± 8.37	51.83 ± 1.10
2‐Methyl‐1‐butanol	44.42 ± 0.24	35.51 ± 0.78	42.95 ± 4.14	52.72 ± 5.43	44.42 ± 0.95
3‐Methyl‐1‐butanol	219.13 ± 3.27	180.26 ± 5.21	222.95 ± 19.62	218.20 ± 23.19	221.86 ± 5.11
2,3‐Butanediol, *levo*	92.80 ± 2.84	74.33 ± 0.72	82.97 ± 10.31	77.30 ± 9.40	76.08 ± 9.57
2,3‐Butanediol, *meso*	32.89 ± 0.27	23.25 ± 0.13	25.21 ± 3.17	23.76 ± 2.35	23.26 ± 2.82
2‐Phenylethanol	42.79 ± 1.65	25.83 ± 0.32	51.04 ± 14.70	33.75 ± 6.15	36.75 ± 4.27

*Note*

The results are shown as the mean values with their standard deviation.

Methanol was also found in blueberry wine at concentrations higher than those typically found in wine (approximately 30–35 mg/L) (Ribéreau‐Gayon et al., 2006). This compound derives from the enzymatic hydrolysis of pectin, and its excessive consumption is potentially harmful. Red wines made by skin contact fermentation in the presence of stems typically contain high levels of methanol, reflecting the variation in skin contact times (Hodson, Wilkes, Azevedo, & Battaglene, 2017). The acceptable limit of methanol in wine is, according to the International Organization of Vine and Wine (OIV), 400 mg/L for red wines and 250 mg/L for white wines and *rosés*. These limits are fixed in the International Code of Oenological Practices (Code) or in the Recueil of International Methods of Analysis for Wines and Musts, limits that are not exceeded in the wines obtained in this study. However, this level can be lowered in blueberry wine by reducing the time of skin contact fermentation or by removing stems that typically contains high levels of pectin. In alcoholic beverages and food fermentations, ester production is the result of hydrolysis reactions catalyzed by esterases, lipases, and alcohol acyl transferases (Costello, Siebert, Solomon, & Bartowsky, 2013). Ethyl acetate and ethyl lactate were the two esters found to have high volatile levels, and their concentrations were similar to those found in wines (Bartowsky & Pretorius, 2009; Genisheva, Macedo, Mussatto, Teixeira, & Oliveira, 2012). The concentrations of acetaldehyde, ethyl acetate, and 1‐propanol were significantly higher in MSF wines than in those obtained under laboratory conditions, while ethyl lactate was lower in the former. Concentrations of 2‐methyl‐1‐propanol, 2‐methyl‐1‐butanol, 3‐methyl‐1‐butanol, and 2‐phenylethanol were independent of the fermenter size or aging.

#### 3.2.2 Minor volatiles

Minor volatile compounds were quantified in blueberry wines by GC‐MS, and the results are presented in Table 4. Esters and terpenic compounds were the groups of volatiles expressed the most in blueberry wines, followed by volatile fatty acids, alcohols, and norisoprenoids. Esters found in fruit wines can derive from either the fruit or sugar fermentation. For instance, ethyl butyrate and ethyl hexanoate have been reported in blueberry fruits (Farneti et al., 2017) but can also be derived via fermentation by yeast, which is responsible for producing several other esters such as isoamyl acetate and ethyl octanoate. Isoamyl acetate, with a banana‐like aroma, ranged from 42.28 to 116.77 μg/L, while ethyl hexanoate and ethyl octanoate, which impart fruity/floral characteristics (Lilly, Lambrechts, & Pretorius, 2000), decreased after aging in bottle, although that decrease was only significant for ethyl octanoate (Table 4). Esters can also derive from chemical esterification of acids when combined with alcohols, which can justify the appearance of ethyl lactate, diethyl malate, and diethyl and monoethyl succinate in the blueberry wines (Bartowsky & Pretorius, 2009). Ethyl esters of succinate, diethyl, and monoethyl, whose descriptors are fruity‐apple or stewed fruit aromas, were the major esters in blueberry wines, and their concentrations were also increased after aging. This result is particularly relevant, given that the formation of ethyl esters of lactic and succinic acid has been considered one of the principal chemical changes during aging, with their contents regarded as indices of the length of aging (Shinohara, Shimizu, & Shimazu, 1979) or as enhancers of fruity characteristics in the alcoholic beverages (Swiegers et al., 2005).

**Table 4:** Concentration of minor volatile compounds (*C*) detected and quantified by GC‐MS in blueberry wines after alcoholic fermentation by the yeast strain *Saccharomyces cerevisiae* QA23 in laboratory‐scale fermentations (LabSFs) and midscale fermentations (MSFs) or after 16 months in bottle

Compounds *C*/(μg/L)	LabSF 1	LabSF 2	MSF 1	MSF 2	Aged in bottle
Ethyl butyrate	nd	74.85 ± 32.46	84.84 ± 5.40	47.41 ± 3.52	79.03 ± 7.88
Isoamyl acetate	116.77 ± 2.11	42.28 ± 2.47	69.19 ± 3.66	50.22 ± 0.82	50.54 ± 1.09
Ethyl hexanoate	119.62 ± 11.42	318.32 ± 15.58	510.08 ± 15.27	321.94 ± 60.41	407.56 ± 20.70
Acetoin	179.33 ± 19.17	111.91 ± 11.54	157.93 ± 21.56	146.96 ± 17.12	120.82 ± 16.22
3‐Methyl‐1‐pentanol	28.6 ± 2.93	52.00 ± 3.04	30.83 ± 0.37	22.35 ± 3.10	34.87 ± 0.52
Ethyl lactate	271.28 ± 5.15	297.75 ± 14.71	95.32 ± 3.49	190.51 ± 33.79	364.73 ± 21.33
1‐Hexanol	77.55 ± 27.76	260.54 ± 9.23	152.99 ± 1.05	663.69 ± 135.35	356.81 ± 16.05
3‐Ethoxy‐1‐propanol	7.23 ± 0.74	17.79 ± 1.10	10.48 ± 0.14	17.53 ± 1.96	51.20 ± 4.10
*Z*‐3‐Hexenol	17.87 ± 7.22	48.24 ± 2.43	39.57 ± 1.19	71.87 ± 15.05	nd
Ethyl octanoate	156.14 ± 15.95	149.82 ± 9.77	156.59 ± 5.31	120.14 ± 28.33	127.03 ± 9.38
*cis*‐Furan linalool oxide	9.23 ± 4.66	6.61 ± 0.51	4.51 ± 0.36	5.56 ± 0.11	71.32 ± 2.42
Linalool	3023.75 ± 399.68	2553.83 ± 60.50	1846.56 ± 33.25	1645.20 ± 215.29	583.50 ± 6.36
2‐Methylpropanoic acid	173.075 ± 16.44	45.63 ± 12.83	46.07 ± 4.04	69.89 ± 7.95	nd
Diethyl malonate	16.92 ± 0.55	10.08 ± 0.01	24.43 ± 0.30	37.61 ± 4.48	36.61 ± 0.46
3‐Methylbutyric + 2‐Methylbutyric acids	246.99 ± 9.57	107.21 ± 1.07	101.17 ± 8.46	125.92 ± 31.83	81.31 ± 25.99
Diethyl succinate	1158.29 ± 86.75	434.13 ± 6.29	1803.56 ± 70.04	2046.35 ± 210.80	5151.20 ± 110.33
α‐Terpineol	1449.28 ± 162.17	1108.19 ± 27.33	751.99 ± 21.04	669.55 ± 69.78	2072.0 ± 92.25
Methionol	38.69 ± 1.73	44.43 ± 15.89	122.33 ± 15.37	154.88 ± 12.95	150.29 ± 13.63
β‐Citronellol	237.53 ± 30.01	392.06 ± 9.77	462.83 ± 0.34	256.12 ± 28.02	68.39 ± 0.05
*trans*‐Carveol	33.67 ± 1.73	12.23 ± 2.74	9.32 ± 0.11	9.39 ± 0.25	31.49 ± 4.60
Hexanoic acid	451.26 ± 33.74	783.78 ± 64.45	714.00 ± 24.54	402.77 ± 59.82	608.05 ± 20.22
Benzyl alcohol	34.83 ± 1.85	125.86 ± 10.72	116.76 ± 0.35	19.43 ± 3.87	77.87 ± 4.69
*p*‐1‐Menthen‐9‐ol	77.02 ± 9.39	98.59 ± 0.89	79.18 ± 3.43	58.59 ± 2.96	181.13 ± 10.83
Linalool hydrate	72.33 ± 14.52	34.27 ± 3.95	19.52 ± 2.48	16.57 ± 1.88	74.26 ± 5.69
Diethyl malate	158.77 ± 3.82	57.04 ± 15.89	34.48 ± 7.23	15.89 ± 3.22	556.53 ± 50.32
Octanoic acid	1013.14 ± 188.30	898.99 ± 34.08	627.77 ± 11.34	370.83 ± 90.56	1760.9 ± 89.18
Eugenol	477.01 ± 66.14	613.46 ± 25.96	539.51 ± 20.10	364.50 ± 39.17	907.51 ± 51.46
4‐Vinylguaiacol	80.02 ± 39.63	53.69 ± 9.02	24.28 ± 7.69	28.59 ± 3.01	69.49 ± 5.10
8‐Hydroxy‐6,7‐dihydrolinalool	106.42 ± 106.56	74.81 ± 7.93	66.54 ± 0.54	25.61 ± 1.82	35.25 ± 3.45
*E*‐8‐Hydroxylinalool	54.21 ± 10.06	42.25 ± 4.63	45.76 ± 4.11	43.01 ± 9.43	59.23 ± 1.01
*Z*‐8‐Hydroxylinalool	582.07 ± 80.27	810.35 ± 103.27	701.09 ± 18.77	412.79 ± 33.18	353.02 ± 5.86
Monoethyl succinate	2300.64 ± 172.82	521.91 ± 26.60	475.29 ± 8.68	502.39 ± 10.72	2374.4 ± 435.49
3‐Hydroxy‐7,8‐dihydro‐β‐ionone	80.16 ± 8.24	29.02 ± 2.18	53.16 ± 7.82	64.63 ± 8.68	79.66 ± 0.24
Methyl vanillate	42.295 ± 6.54	51.75 ± 2.43	48.43 ± 7.57	45.91 ± 1.10	44.68 ± 0.81
3‐oxo‐α‐ionol	13.78 ± 0.97	31.09 ± 4.57	24.85 ± 2.25	19.43 ± 4.25	30.29 ± 1.07
3‐Hydroxy‐7,8‐dihydro‐β‐ionol	48.04 ± 2.61	16.99 ± 1.68	27.81 ± 0.18	24.00 ± 2.70	53.64 ± 0.18
Syringaldehyde	1054.44 ± 148.46	524.09 ± 148.60	138.65 ± 47.44	nd	nd

*Notes*

The results are shown as the mean values with their standard deviation.

nd: not detected.

Terpenic compounds found in blueberry wines were also diverse. Several terpenic compounds had been previously reported in blueberry fruits, namely linalool, linalool oxide, and α‐terpineol, were identified (Beaulieu, Stein‐Chisholm, & Boykin, 2014; Du & Rouseff, 2014; Farneti et al., 2017). The evolution of terpenes during aging is of particular importance for understanding their content in blueberry wines. The linalool concentration decreased significantly during aging, probably due to chemical phenomena, especially oxidation reactions. During fermentation, certain enzymes of *S. cerevisiae* have been described to cyclize linalool to α‐terpineol (Takoi et al., 2010), which is evident due to the increase in its concentration. As a result of the high linalool content, several other related compounds were found in blueberry wine, which can have varietal origins; these compounds include 8‐hydroxylinalool, whose concentration did not vary with aging but can be affected by reactions involving linalool; for example, the concentration of linalool hydrate increased significantly during storage. Additional terpenic compounds such as *trans*‐carveol, β‐citronellol, and *p*‐1‐menthen‐9‐ol were found in blueberry wines, but these compounds can also originate from the fruit due to its terpenoid composition. Among these compounds, β‐citronellol concentration decreased significantly during aging and is believed to be correlated with the degradation of this compound because it is prone to oxidation by light or hydrogen peroxide (Komaszylo née Siedlecka et al., 2016).

Medium‐chain fatty acids (MCFAs) are produced through lipid metabolism by yeasts and are usually associated with unpleasant aromas such as fatty, sweaty, rancid, or cheese aromas (Ferreira, López, & Cacho, 2000). In this study, octanoic acid was the only MCFA present in all wines, but it was present in significantly different concentrations, ranging from 0.58 mg/L (in wines before aging in bottle) to 1.76 mg/L (in wines after aging in bottle).

Two volatile phenols were found in blueberry wines, namely eugenol and 4‐vinylguaiacol. Eugenol can also be of a varietal origin, having been reported in blueberry cultivars (Du, Plotto, Song, Olmstead, & Rouseff, 2011; Du & Rouseff, 2014; Farneti et al., 2017). In grape berries, 4‐vinylguaiacol is present as glycosylated precursors; thus, it can be speculated that something similar occurs in blueberry. This compound can be produced from ferulic acid, a natural constituent of berries, by the activity of hydrocinnamate decarboxylase, which converts it to 4‐vinylguaiacol (Boulton et al., 1996). In blueberry wines, 4‐vinylguaiacol was detected in concentrations ranging from 24.28 to 80.02 μg/L, and these levels are below the threshold value of 426 μg/L (Chatonnet, Dubourdieu, Boidron, & Pons, 1992); exceeding this level leads to unpleasant mousy, animal, horsy, barnyard, smoky, spicy, or medicinal phenolic character (Heresztyn, 1986; Chatonnet et al., 1992).

Lastly, norisoprenoids were found in blueberry wines, namely 3‐hydroxy‐7,8‐dihydro‐β‐ionone, 3‐oxo‐α‐ionol, and 3‐hydroxy‐7,8‐dihydro‐β‐ionol. These compounds derive mainly from breakdown of carotenoids and are of varietal origin, having previously been identified in grapes (Mendes‐Pinto, 2009). Therefore, norisoprenoids found in blueberry wines are believed to be of varietal origin.

**Figure 2:**
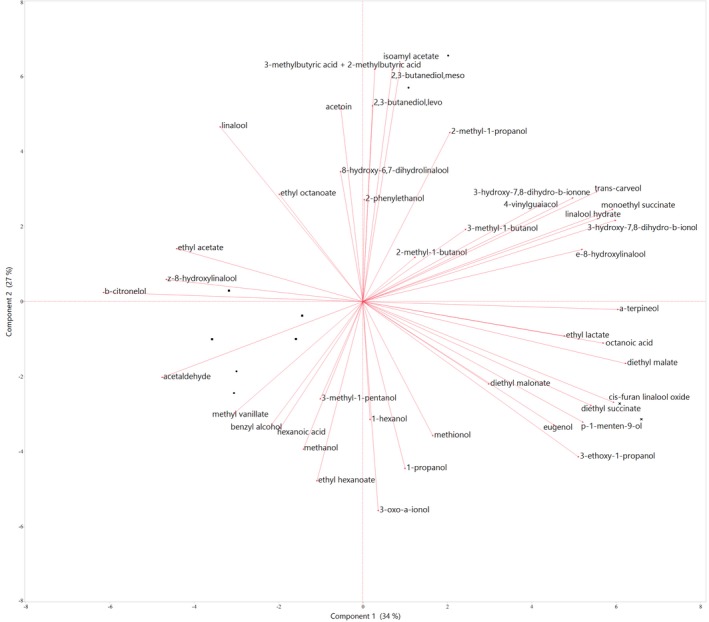
Two‐dimensional plot from principal component analysis based on the concentrations of volatile aroma compounds identified and quantified by GC‐MS (for minor compounds) and GC‐FID (for major compounds) in blueberry wines after alcoholic fermentation by the yeast strain *Saccharomyces cerevisiae* QA23 in laboratory‐scale fermentations (LabSFs) and midscale fermentations (MSFs) or after 16 months in bottle. ● LabSF; ■ MSF;** X** Aged in bottle

To obtain a more abridged view of the relationship among blueberry wines and their aroma composition, PCA was applied to all data obtained (Table 3 and Table 4). This approach allowed the identification of the compounds that better discriminated the wines. The first two principal components accounted for 61.5 % of the total variance. PC1 accounted for 34.5 %, and PC2 accounted for an additional 27 % of the total variability (Figure 2). Wines obtained under LabSF and MSF conditions are in the left lower quadrant, with the exception of one replicate (LabSF 1) allocated on the upper right quadrant, creating a distinctive group characterized by low concentrations of acetaldehyde, methanol, 1‐hexanol and ethyl hexanoate, high concentrations of 2‐methyl‐1‐propanol, isoamyl acetate, and methylbutyric and the production of polyalcohols such as 2,3‐butanediols. This experiment contrasts with the others that we performed because it was the only experiment where no adjustments of *YAN* were performed; the initial concentration of 126 mg/L was considered sufficient for the yeast to complete alcoholic fermentation. Early studies showed that the type of nitrogen source affects yeast growth and metabolites produced by yeast (Barbosa, Mendes‐Faia, & Mendes‐Ferreira, 2012; Webster, Edwards, Spayd, Peterson, & Seymour, 1993). The wines positioned in the lower right quadrant correspond to the wines that experienced 16 months of aging in bottles and are highly loaded with the esters, diethyl succinate, ethyl lactate (ethyl 2‐hydroxypropanoate), and diethyl malonate. PCA also showed that the global aroma of the wines could separate them into two well‐defined quadrants (aged and nonaged wines) and a third group separated by differences in the initial nitrogen source.

## 4 CONCLUSIONS

This work reveals that the fruit wines obtained from blueberry present a set of compounds that usually arise from unmodified fruit. Indeed, several terpenic compounds have been identified, including linalool, *cis*‐furan linalool oxide, α‐terpineol, *trans*‐carveol, β‐citronellol, and *p*‐1‐menthen‐9‐ol. The presence of nor isoprenoids, namely 3‐hydroxy‐7,8‐dihydro‐β‐ionone, 3‐oxo‐α‐ionol, and 3‐hydroxy‐7,8‐dihydro‐β‐ionol, appears to be a varietal characteristic of this fruit. A set of other compounds came directly from the metabolic activity of yeast; this set can be called the “fermentation bouquet” as it is in wine, and it is assumed to account for the aroma and mouthfeel perception. This fermentation bouquet consists of alcohols, esters, acids, and carbonyl compounds, which are typical of an alcoholic beverage and mostly contribute positively to wine quality. Interestingly, the levels of some of the alcohols in this fruit wine were above the described perception threshold (2‐methyl‐1‐butanol, 3‐methyl‐1‐butanol, and 2‐phenylethanol), and even acetaldehyde surpassed the concentrations usually found in wines. Esters of succinate, generally associated with aging, increased in blueberry wines after 16 months of aging in bottles. Nevertheless, further study is still needed to confirm the sensory contributions of the panoply of the volatile compounds identified in blueberry wines. The results obtained anticipate the potential of this fermented beverage in a market where innovation and new products are increasingly sought by consumers.

## CONFLICT OF INTEREST

The authors declare no conflict of interest.

## ETHICAL STATEMENT

This article does not involve any studies with human participants or animal testing​ performed by any of the authors.
